# Salinity stress drives herbivory rates and selective grazing in subtidal seagrass communities

**DOI:** 10.1371/journal.pone.0214308

**Published:** 2019-03-21

**Authors:** Sahira Y. Bell, Matthew W. Fraser, John Statton, Gary A. Kendrick

**Affiliations:** 1 School of Biological Sciences and UWA Oceans Institute, Faculty of Science, University of Western Australia, Crawley, Western Australia, Australia; 2 Balu Blue Foundation, Port Lincoln, South Australia, Australia; Mississippi State University, UNITED STATES

## Abstract

The role of environmental-stress gradients in driving trophic processes like grazing, has potential to shape ecosystem responses to environmental change. In subtidal seagrass systems, however, the variation in top-down processes along stress gradients are poorly understood. We deployed herbivory assays using the five most common seagrass species of Shark Bay, to determine whether herbivory pressure changed across a salinity-stress gradient from oceanic (38 PSU) to hyper-saline (51 PSU) conditions. Seagrass tissue removed from herbivory assays by fishes decreased as environmental stress increased, and herbivores consumed greater amounts of tropical seagrass species compared to the temperate species that dominate seagrass cover in Shark Bay. This heightened consumption was correlated with enriched seagrass nutrient concentrations. Our work suggests there’s a fundamental relationship between trophic interactions and environmental conditions within complex marine settings. Abiotic stressors like salinity directly impact seagrass communities physiologically; however we show that salinity stressors also shift biotic interactions, indirectly influencing grazing rates and thus having a greater effect on seagrasses than physiological impacts alone. In Shark Bay where restoration efforts are being employed to address large scale loss of seagrasses, the relationship between herbivory pressure and salinity-stress could therefore prove crucial to restoration success.

## Introduction

Top-down control can be useful in understanding herbivory interactions within macrophyte communities [1], particularly when extreme environmental conditions vary within a region. As multiple factors such as light, temperature, nutrients or salinity (bottom-up) and competition and herbivory (top-down) can influence the structure and dynamics of macrophyte communities, and it can therefore be difficult to interpret the dominant drivers of community interactions. Plant-herbivore trophic interactions are highly influential in structuring marine systems, with shifts in the herbivore composition due to environmental stress (e.g. temperature, salinity) having the capacity to severely impact ecosystem function [2–4]. Accurate predictions of marine ecosystem responses to environmental change therefore rely upon an understanding of species interactions (direct and indirect) in combination with the influence of abiotic factors.

Grazing by herbivorous fish is a fundamental ecological process structuring seagrass ecosystems [5–7], yet the environmental drivers that influence grazing rates and grazer abundance are relatively understudied. Herbivory rates are influenced by a variety of factors, many of which are also influenced by changes in salinity. For example, macrophyte availability, habitat heterogeneity, leaf texture and chemical composition [8–12] influence the rate of herbivory, and can themselves be impacted by salinity changes. Fish consumers’ forage choice also plays a vital role in regulating the coexistence of multiple seagrass species within a trophic level [11]. Herbivores preferentially graze on seagrass species with higher nutrient content [5, 13]; however, results vary among species and spatially within species [14]. As a result, the distribution and abundance of seagrass communities are controlled when species of higher nutrient content are preferentially grazed, meaning less palatable species become most abundant [11]. From such studies it is clear that feedbacks in grazed ecosystems can influence seagrass species composition [15]. Therefore, quantifying how environmental factors regulate herbivore grazing rates is central to understanding system dynamics of seagrass ecosystems [16].

Many studies that have incorporated seagrass-consumer interactions to date have been conducted in tropical seagrass environments or in systems heavily impacted by anthropogenic processes [17–21], with fewer applied to pristine systems with a mix of temperate and tropical species [22]. Shark Bay, Western Australia represents an ideal location to develop a predictive framework for changes in herbivory interactions within an environment exposed to minimal anthropogenic impacts, a strong environmental gradient (salinity) and containing intact herbivore communities. Shark Bay is located at an overlap of biotic provinces where ecological communities within the area have both tropical and temperate members, contributing to its high biodiversity. In Shark Bay, a strong, semi-permanent hyper-salinity gradient is present throughout the embayment [23] and is maintained by high rates of evaporation and limited water exchange with adjacent oceanic waters. This salinity-stress gradient (ranging between 36 PSU and 65 PSU) is a permanent feature, and results in physiologically stressed seagrasses in hyper-saline areas [23]. As such, it is likely to have a substantial influence over seagrass community dynamics, including grazing pressure [24–26].

Here, we examined changes to the top-down influence of herbivory pressure and feeding choice on seagrasses, across the permanent salinity gradient from normal oceanic levels (38 PSU) to hyper-saline conditions (51 PSU) in Shark Bay, Western Australia. Our hypotheses were; (i) the total biomass of seagrass removed from herbivory trials would decrease as salinity increased and (ii) the fast growing, small-bodied, tropical seagrass species that are high in nutrients would be selected by grazers.

## Methods

### Site description

**Fig 1:**
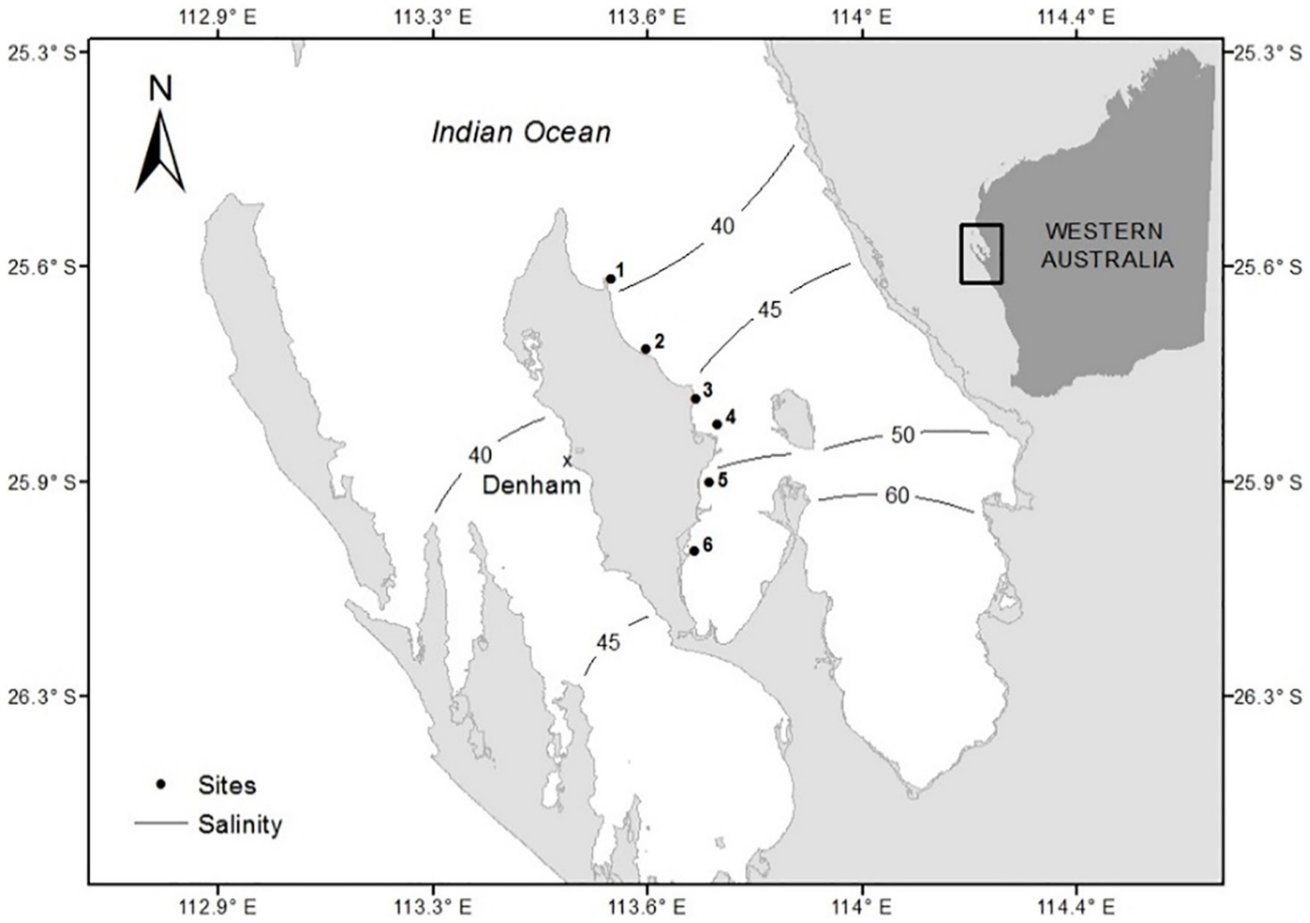
Location of six study sites and coastal town of Denham in relation to the salinity gradient in Shark Bay, Western Australia. Sites recorded the following average salinities *in situ* (north to south): 1 = 38 PSU, 2 = 39 PSU, 3 = 41 PSU, 4 = 44 PSU, 5 = 48 PSU and 6 = 51 PSU. Salinity contour lines are redrawn from Walker (1985) and are represented by solid black lines. Insert shows the location of Shark Bay relative to the rest of Western Australia.

Shark Bay is 13,000 km^2^ in size and is located ~800 km north of Perth, Western Australia (25°47’S, 113°43’E, Fig 1). Salinity ranges from 36 PSU at the northern embayment opening to >65 PSU in the southern Hamelin Pool region [27]. Shark Bay is a shallow (<15 m), relatively isolated, large subtropical coastal embayment containing some of the most species rich seagrass meadows in the world [23]. Seagrasses are the dominant benthic community covering an estimated 3500 km^2^ [28] and contribute to its listing as a UNESCO World Heritage Area [26]. Meadows are dominated by the larger temperate seagrasses *Amphibolis antarctica* and *Posidonia australis*, that typically form dense and monospecific canopies [23]. Smaller, faster-growing, tropical to subtropical *Halophila ovalis*, *Halodule uninervis*, and *Cymodocea angustata* are also common but found in much lower abundances, as mixed meadows, understory or as a single species patch in extreme environments (Table 1) [23].

**Table 1:** Summary of the extent of the five most abundant seagrasses of Shark Bay, Western Australia, to a range of environmental conditions. Table has been adapted from Walker et al. (1988).

Species	Depth (m)	Salinity (‰)	Notes
*Posidonia australis*	0–14	35–55	On bands and channels
*Amphibolis antarctica*	0–15	32–62.4	Found under all conditions
*Cymodocea angustata*	0–14	38–50	In sand patches or as understory
*Halodule uninervis*	Intertidal-14	35–64	In sand flats or as understory
*Halophila ovalis*	Intertidal-14	35–55	On sand flats or edge of banks

### Herbivory assays

**Fig 2:**
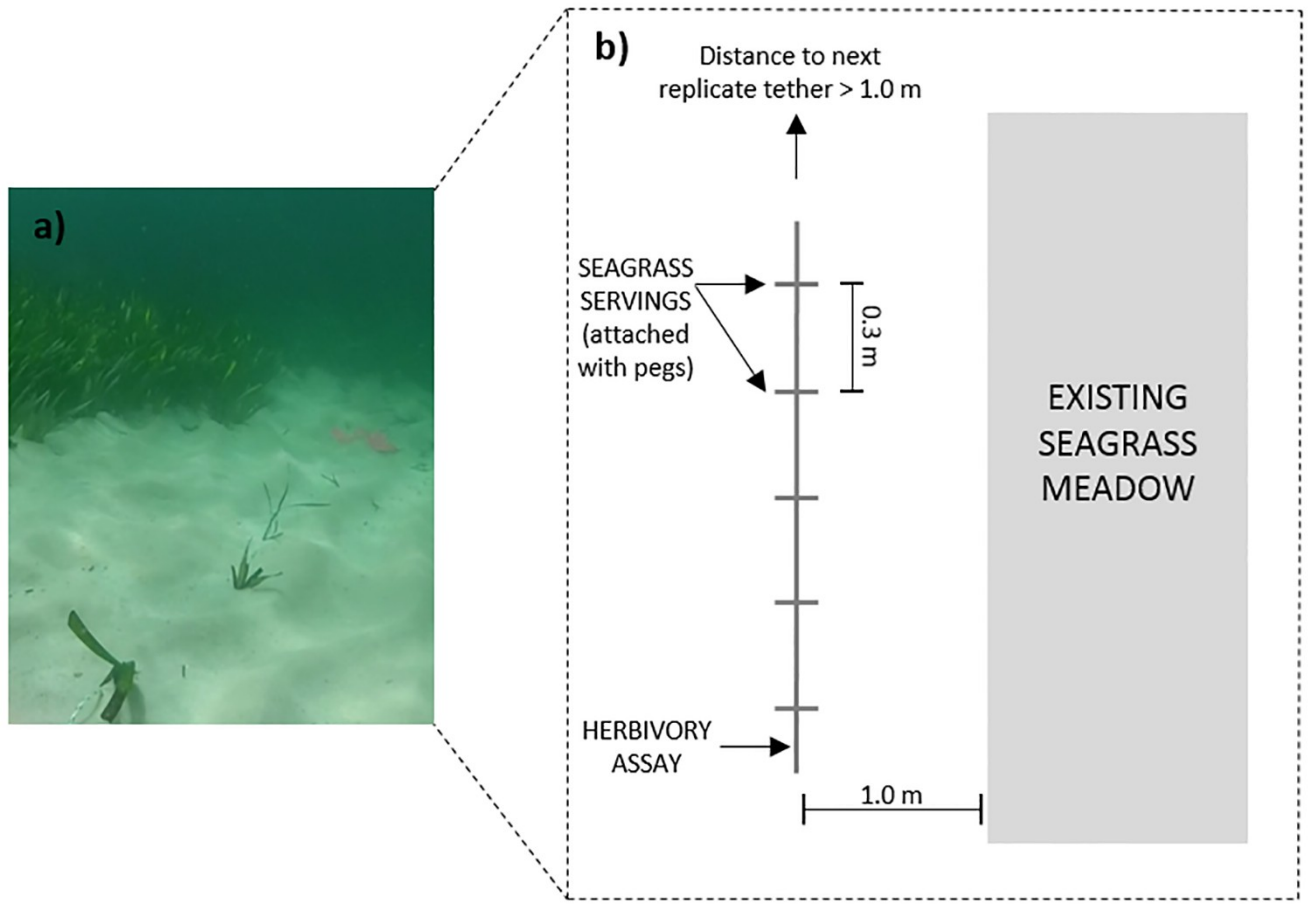
(a) Image of a deployed herbivory assay. NB: that pink flagging tape observed in the image was removed after assay deployment. Photo credit: Sahira Bell. (b) Schematic representation of herbivory assay deployment *in situ*. Schematic is not to scale.

**Fig 3:**
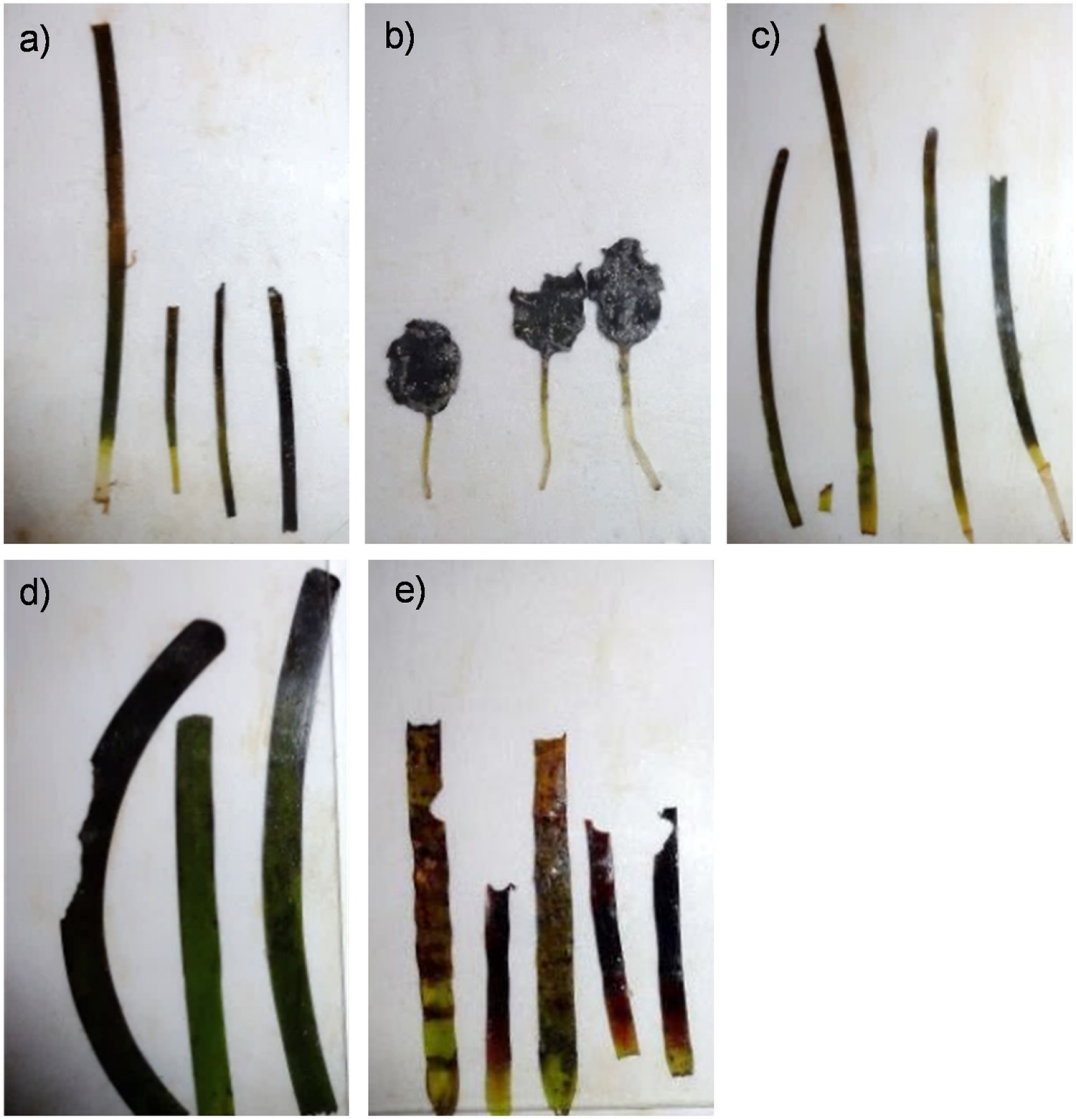
(a) *Halodule uninervis*, (b) *Halophila ovalis*, (c) *Cymodocea angustata*, (d) *Posidonia australis* and (e) *Amphibolis antarctica* leaves with signs of herbivory after being deployed for a period of 24 hrs. Images are not set to the same scale for bite mark clarity. Photo credit: Sahira Bell.

Herbivory pressure was assessed using herbivory assays (tether deployments, Fig 2) comprised of the five most abundant seagrass species; *A*. *antarctica*, *P*. *australis*, *H*. *uninervis*, *H*. *ovalis* and *C*. *angustata* (Fig 3). Six study sites within the eastern embayment ranging from Guichenault Point to L’haridon Bight were identified for assay deployment to sample across the strong salinity gradient (Fig 1). Sites were selected to keep tidal influence, current strength, sediment type and temperature consistent [29–31]. At each site, six forage-choice assays were deployed at least 1 m apart in bare sand, within 1 m of an existing seagrass meadow, between depths of 3–5 m (Fig 2). Bare sand was chosen for deployment in order to allow the assays to be distinguished from the natural seagrass patches seen in the camera recordings. Similar topographic landscape was targeted for assay deployments, as surrounding landscape can impact a variety of ecological processes [32, 33]. Five sites were sampled (n = 30 assays) in March (summer: sites 1,2,3,4 and 6) and in July 2014 (winter: sites 1,2,3,5 and 6). Site 4 was not sampled in winter, instead it was replaced with site 5 as to target a higher salinity and gain a more thorough representation of the salinity gradient. In preparation for forage-choice assay deployment fresh seagrass leaves were collected (removed from their rhizomatic parts) from a donor seagrass meadow the evening prior and stored in seawater (~39 PSU) until deployment. Attributes of seagrass donor sites were kept consistent (i.e. different seagrass patches to negate depletion of an area, but consistent salinities targeted). On each assay three leaves were used for *P*. *australis* servings, and five leaves were used for *A*. *antarctica*, *C*. *angustata*, *H*. *ovalis* and *H*. *uninervis* servings in order to approximately standardise for biomass [22]. Only clean, undamaged leaves with no evidence of grazing marks or epiphytes were used for each trial. Each serving was attached to a 3mm nylon line, with servings spaced every 30 cm along the line. In order to simulate live seagrass, the servings were secured to the line with pegs to stand vertically with the nylon rope secured to the sea floor using metal tent pegs. All five seagrass species were present on each forage choice assay, with the species order being randomly assigned.

Total biomass removed (%) due to herbivory was assessed using digital photographs. Prior to a deployment each seagrass serving was photographed individually with a tag indicating the site and assay number. Each forage-choice assay was deployed for 24 hrs, before being collected and re-photographed and analysed using the area measurement function in the program CPCe [34]. The initial measurement of leaf area was subtracted from the leaf area remaining after 24 hours then converted to biomass lost due to herbivory based on the biomass constant of the species (using conversion: dry weight * area lost).

Discrete video cameras were deployed at each site to ensure seagrass leaf loss was due to herbivory and not due to any leaf damage caused by handling or deployment, and to identify grazers. Go-Pro Hero2/3+ cameras were set in custom-built underwater housings, mounted on a 10cm pole and positioned at an assay’s corner focussing on the seagrass servings. The camera was positioned to enable all five seagrass species to be observed in a single frame. With the aid of battery pack extensions each camera had an average recording time of ~3hrs.

Salinity was recorded at each site using a YSI CastAway-CTD (YSI Environmental 2010, Ohio, USA). CTD drops were made prior to assay deployment and repeated once all assays were in place (approximately 1 hour later) to take into account the fluctuations of salinity within a tidal cycle. CTD drops were conducted in summer and winter, with average salinity for each site determined from these values.

### Nutrient content analysis

Seagrass samples (n = 30 leaves) were collected from the donor beds in summer and winter for nutrient content analysis. Leaves from mature specimens of each species were collected. All samples were stored on ice then immediately frozen upon return to shore, where they remained frozen until processing.

Seagrass leaves were thawed and oven-dried at 60°C, then ground to a fine powder with a ball and mill. Carbon (C) and Nitrogen (N) concentrations, C:N ratios and *δ*^13^C and *δ*^15^N isotope signatures were determined from subsamples of seagrass in both winter and summer samples using an Automated C/N Analyser-Mass Spectrometer consisting of a 20/22 mass spectrometer connected to an ANCA-SI preparation system (SERCON, UK) at the Western Australian Biogeochemistry Centre, the University of Western Australia. All samples were standardised using a multipoint normalisation against a secondary reference of Radish collegate (3.167% N, *δ*^15^N 5.71%, 41.51% C, *δ*^13^C 28.61%). This was in turn standardised against primary analytical standards (International Atomic Energy Agency (IAEA) Vienna) [35, 36]. For estimates of the C:N ratio, the external error of analysis (one standard deviation) did not exceed 0.1. For *δ*^13^C and *δ*^15^N calculations error was no more than 0.1‰ and 0.2‰ respectively.

This study was completed under permit number SW016069 from the WA Department of Biodiversity, Conservation and Attractions. All data are available online through the figshare repository (10.6084/m9.figshare.3506276).

### Statistical analysis

A conditional approach to analyses was used due to the large number of zeros in the dataset. A linear mixed effects model in the statistical package R (version 3.1.2, nlme package [37, 38]) was used to investigate factors impacting the proportion of leaf biomass removed by consumers. Data were square root transformed in order to meet assumptions of normality, and were analysed for effects of Species, Season and Salinity, with Site included as a random factor. Interactions of the model were also investigated. Where interactions were significant, Tukey’s Honest Significant Difference (HSD) tests were conducted to further explore the effects. To explore the role of seagrass nutrient content in the context of herbivory pressure, analysis of variance (ANOVA) was used to determine differences in nutrient content between seagrass species and seasons. To further explore the correlation between nutrient content and grazing pressure, Pearson correlation tests were employed.

## Results

### Herbivory assays

**Fig 4:**
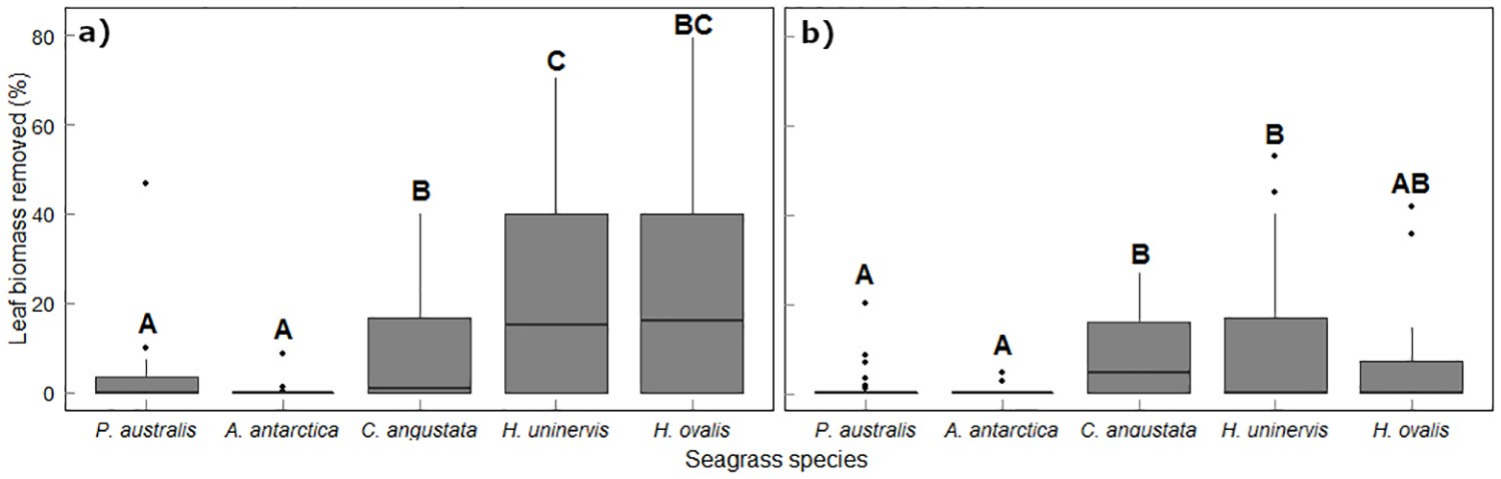
Proportion of leaf biomass removed in summer (left) and winter (right) for the five most common seagrass species of Shark Bay, Western Australia. Seagrass species are arranged along the x-axis according to leaf turnover rates from the slowest to the fastest species. Median (horizontal line), first and third quartile (hinges) and 95% confidence intervals (notches) are shown. Letters correspond to significant differences in biomass removed (Tukey HSD).

**Fig 5:**
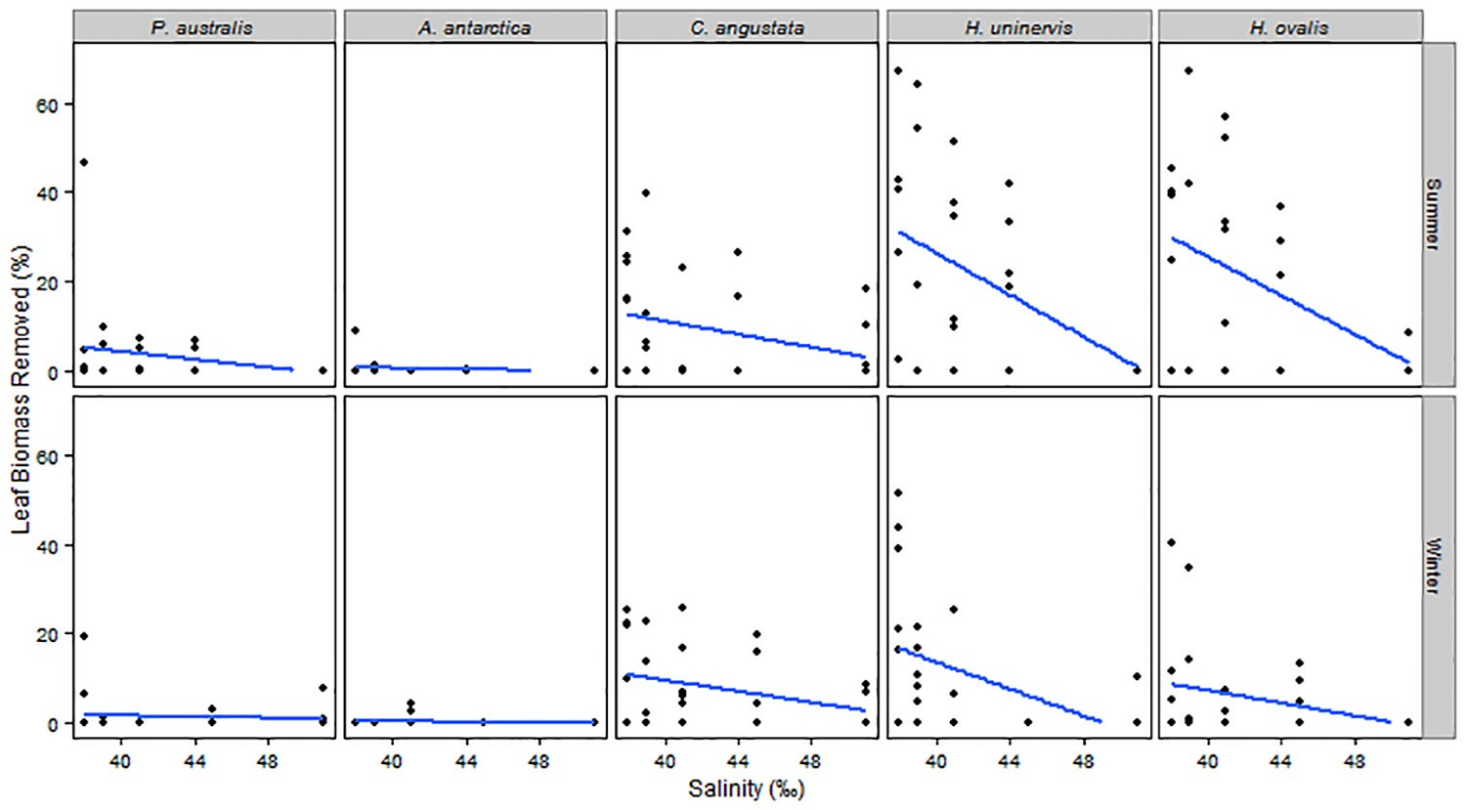
Seagrass leaf biomass removed (%) during summer (top) and winter (bottom) for the five most common seagrass species of Shark Bay, Western Australia. Lines (blue) represent linear regression.

Total leaf biomass removed by consumers ranged from 55.8 g (dry weight) at the lowest salinity (38 PSU) to 3.07 g at the highest salinity (51 PSU). The proportion of leaf biomass removed from seagrass servings varied significantly across the salinity gradient (*P* = 0.001, Table 2), however this was not consistent across Species, with a significant Salinity by Species interaction (*P <* 0.001, Table 2). The proportion of biomass removed decreased as the environmental stress of salinity increased, with greater removal rates observed only for the tropical species *Cymodocea angustata*, *Halodule uninervis* and *Halophila ovalis* (Figs 4 and 5). There was significant variation in the proportion of leaf biomass removed between Species, however this was not consistent across both Seasons, with a significant Species by Season interaction (*P* < 0.004, Table 2, Fig 4). Post Hoc tests revealed that in summer, *H*. *uninervis* and *H*. *ovalis* experienced significantly higher grazing rates than the temperate seagrass species. *Cymodocea angustata* showed intermediate grazing rates, with post hoc tests revealing a significant difference observed only in comparison to *H*. *ovalis* (Fig 4a). In winter, the only significant difference observed was between grazing rates of *H*. *uninervis* and *Posidonia australis* (Fig 4b). When examining species differences across seasons, only *H*. *ovalis* showed significant change, with a higher proportion of leaf biomass removed in summer than winter.

**Table 2:** Results of ANOVA examining the total leaf biomass removed in summer and winter in response to Salinity (Sa), Species (Sp) and Season (Se). Significant *P* values are highlighted in bold.

Factor	*df*	F-value	*P value*
Salinity	5	77.25	**0.001**
Species	4	16.25	**<0.001**
Season	1	21.74	**0.006**
Sa x Sp	17	11.92	**<0.001**
Sa x Se	3	7.09	0.06
Sp x Se	3	3.63	**<0.01**
Sa x Sp x Se	5	3.92	0.619

### Nutrient content of seagrass species

There was significant variation among seagrasses in C:N ratios (*P* = 0.02), but not in C:P ratios (*P* = 0.07). The faster growing tropical seagrass species had higher N and P concentrations than the temperate *Posidonia australis* and *Amphibolis antarctica* (Table 3). *Cymodocea angustata* had the highest relative N content (i.e. lowest C: N ratio), with *H*. *uninervis* and *H*. *ovalis* showing similar and intermediate values. Nitrogen concentrations were lowest in *P*. *australis* and *A*. *antarctica*. Similarly, P concentrations were also lowest in *P*. *australis* and *A*. *antarctica* and highest in *H*. *ovalis* (Table 3). *Cymodocea angustata* and *H*. *uninervis* had similar and intermediate values. Pearson correlation tests revealed a significant correlation between C:N ratio and grazing intensity (*P* = 0.04, correlation coefficient -0.65), but no significant correlation between C:P ratio and grazing intensity (*P* = 0.37, correlation coefficient -0.33). No significant difference in nutrient content was detected between seasons (Nitrogen *P* = 0.29, Phosphorus *P* = 0.15).

**Table 3:** Average nutrient content by weight for each of the five sampled seagrass species. Note that lower nutrient ratio values indicate species of greater quality i.e. higher relative N or P content.

Species	C:N (weight)	C:P (weight)
*Posidonia australis*	20.4	311.5
*Amphibolis antarctica*	23.2	373.9
*Cymodocea angustata*	14.6	213.9
*Halodule uninervis*	18.4	231.6
*Halophila ovalis*	17.6	126.3

### Video analysis

A total of 120 hrs of video footage was captured across both sampling periods. This accounted for 8.3% of total assay deployment time being recorded. Four direct herbivory events were observed (one in summer, three in winter), and there were no patterns associated with these events. Consumers included the herbivorous rabbitfish (*Siganus fuscescens*) and the Western striped grunter (*Helotes octolineatus–*previously *Pelates octolineatus*), which were both observed to consumer *P*. *australis* and *C*. *angustata* servings. Consumption ranged from 0.16 cm^2^ to 3.3 cm^2^. A comprehensive list of all fish species recorded within the Eastern embayment of Shark Bay is provided as supplementary material (S1 Table).

## Discussion

We show that increasing salinity in Shark Bay correlates to changes in the biotic interaction of grazing, indirectly influencing seagrass biomass. Leaf biomass removed by grazing fish decreased as the environmental stress of salinity increased (Fig 5), supporting our first hypothesis and highlighting salinity as a potential driver of seagrass herbivory in Shark Bay. Abiotic stressors like salinity directly impact seagrass communities growth rates and distribution [39, 40]; however here we demonstrate that salinity also reduces the rate of top-down interactions, indirectly influencing seagrass meadows. Extreme salinity stress for seagrasses is a predominant feature of Shark Bay [26], and salinity influences seagrass standing stock in two ways; directly through physiological stress [27], and indirectly through the top-down impact of grazing. This study underscores the importance of integrating abiotic and biotic processes when studying community interactions and advances our understanding of utilising tethering techniques to do so.

The hypothesis that fast-growing, small bodied tropical seagrass species with higher N and P contents would be grazed more heavily was supported, despite these species having a lower abundance and biomass across Shark Bay [23]. The least grazed species (*Amphibolis antarctica* and *Posidonia australis*, Fig 4) are the most abundant species in Shark Bay, typically forming large, dense and monospecific canopies [23]. As such, they provide a key ecological service as structurally complex habitat, further facilitating herbivory on the smaller, nutrient enriched tropical species [22, 41]. Sediments within Shark Bay are oligotrophic with sediment P concentrations extremely low across the study area [42, 43]. As a result, seagrass species relatively enriched in nutrients are generally found as mixed meadows, understory or as a single species patch in extreme environments (up to 64 PSU, Table 3) [23]. Interestingly, it appears that consumers within this system are willing to target rarer and smaller seagrass species with higher nutrient concentrations, despite them being harder to locate across the salinity gradient and containing substantially smaller biomass. Seagrass meadows are commonly considered as habitat providing structures rather than a food source [44, 45]. However, we show substantial consumption of seagrass leaves over small time scales (24 hrs), contrasting studies suggesting seagrass herbivory is infrequent [46–48]. These findings highlight the importance of seagrasses in nutritionally supporting the ecosystems in Shark Bay, however we also acknowledge there are additional seagrass qualities (i.e. morphological defences–fibre content [49], structural carbohydrates [50]) not accounted for that may also be of influence.

All herbivores are not influenced by salinity in the same way, therefore grazer type may also affect forage choice [10]. Due to camera availability and battery life limitations in the video component of this study, we were unable to draw quantitative conclusions as to which mesograzers were responsible for seagrass consumption. The observed shift in preferential grazing between seasons was most likely attributed to changes in consumer communities [51], however this again cannot be concluded from this study alone. Nevertheless, it is interesting to note the presence and observed feeding of the striped trumpeter (omnivore: *Helotes octolineatus*) and rabbitfish (herbivore: *Siganus fuscescens*) within this study; a herbivore which has been documented in Shark Bay on only two other occasions [52, 53] despite extensive fish surveys conducted in the area [54–57]. Distribution of *S*. *fuscescens* down the West Australian coast has extended poleward in recent years due to warming ocean waters [58], with associated herbivory from this species proving catastrophic for macrophyte communities [59]. With ocean temperatures and marine heatwaves predicted to increase in the coming years [3, 60], the capacity for Shark Bay to withstand associated increases in herbivore communities will likely be tested. As such, our study’s comprehensive baseline record of herbivory pressure and identification of consumers will be crucial in underpinning future mechanisms of impact, given that these abiotic impacts can prove detrimental to seagrass communities [61].

Tethering techniques employed by this study have proved an effective tool to quantify the impact of seagrass herbivory, however this technique does not come without its limitations. For example, herbivory assays were placed within bare sand ~1m from an existing seagrass meadow in order to be easily located and retrieved, and filmed. Herbivory pressure within seagrass meadows is not equal (Johns refs 2, 40, 41), and other studies specific to Shark Bay have revealed a greater pressure on seagrasses at a meadows edge as opposed to those within the meadow [62]. As such, this study takes great care in interpreting absolute values of herbivory pressure and instead focuses on the change in pressure across the gradient. Despite this limitation, the *in situ* tethering technique provides strong evidence for changes to seagrass herbivory in Shark Bay, enabling us to examine both abiotic and biotic variables impacting seagrass communities.

Environmental stress has the capability to directly and indirectly influence foundation communities, completely transforming the benthic structure of marine ecosystems [2, 60, 63]. By examining species interactions in combination with abiotic factors this study suggests that salinity is a key environmental driver of herbivory pressure in Shark Bay. We contribute to the wealth of new evidence demonstrating the importance of top-down control in seagrass ecosystems, and highlight the importance of accounting for herbivory when understanding macrophyte dynamics. For Shark Bay; an area which has recently seen dramatic losses of seagrass meadows due to abiotic stress and physical disturbances, this relationship between herbivory pressure and salinity-stress could prove crucial to restoration success and future management of this UNESCO World Heritage region.

## Supporting information

S1 Table Comprehensive list of fish species found in the Eastern embayment of Shark Bay, Western Australia. Table has been adapted from Travers and Potter (2002), Jackson et al. (2007), Belicka et al. (2012), Heithaus et al. (2012) and Walker et al. (2012).
(DOCX)
