# Supplementary material for: Salinity stress drives herbivory rates and selective grazing in subtidal seagrass communities
Source: PLoS One. 2019 Mar 21;14(3):e0214308. doi: 10.1371/journal.pone.0214308 (PMC6428295; doi:10.1371/journal.pone.0214308)
Supplement: S1 Table — Table has been adapted from Travers and Potter (2002), Jackson et al. (2007), Belicka et al. (2012), Heithaus et al. (2012) and Walker et al. (2012). (DOCX) [file pone.0214308.s001.docx]

**Supporting information for the following article**

**Title**

Environmental stress drives herbivory rates and selective grazing in subtidal seagrass communities

**Journal;** Plos One

**Authors**; Sahira Y. Bell^1,2*^, Matthew W. Fraser^1^, John Statton^1^, Gary A. Kendrick^1^

^1^School of Biological Sciences and UWA Oceans Institute, Faculty of Natural and Agricultural Science, University of Western Australia, Crawley, 6009, Western Australia, Australia

^2^ Balu Blue Foundation, Port Lincoln, 5606, South Australia, Australia

*Corresponding author:
Email: sahira.bell@research.uwa.edu.au
Phone: (+61 8) 6488 5813

**S1 Table.** **Comprehensive list of fish species found in the Eastern embayment of Shark Bay, Western Australia. Table has been adapted from Travers and Potter (2002), Jackson et al. (2007), Belicka et al. (2012), Heithaus et al. (2012) and Walker et al. (2012).**

| Species | Year Published | Reference |
| --- | --- | --- |
| Acanthopagrus latus | 2007 | Jackson et al. 2007 |
| Amniataba caudavittata | 2007, 2011, 2012 | Walker et al. 2012, Jackson et al. 2007, Belicka et al. 2012, Heithaus et al. 2011 |
| Apogon rueppellii | 2002, 2007, 2011, 2010 | Walker et al. 2012, Travers and Potter 2002, Jackson et al. 2007, Heithaus et al. 2011 |
| Apogon victoriae | 2002 | Travers and Potter 2002 |
| Atherinomorus vaigiensis | 2011 | Heithaus et al. 2011 |
| Carcharhinus cautus | 2012 | Walker et al. 2012 |
| Centrogenys vaigiensis | 2002, 2007, 2011 | Travers and Potter 2002, Jackson et al. 2007 |
| Colurodontis paxmani | 2011 | Heithaus et al. 2011 |
| Congrogadus subducens | 2012 | Walker et al. 2012 |
| Choerodon cauteroma | 2002 | Travers and Potter 2002 |
| Choerodon cyanodus | 2002 | Travers and Potter 2002 |
| Craterocephalus mugiloides | 2011 | Heithaus et al. 2011 |
| Cymbacephalus nematophthalmus | 2012 | Walker et al. 2012 |
| Echeneis naucrates | 2007 | Jackson et al. 2007 |
| Glaucostegus typus | 2012 | Walker et al. 2012 |
| Hypopterus macropterus | 2002 | Travers and Potter 2002 |
| Inegocia japonica | 2002 | Travers and Potter 2002 |
| Labracinus lineatus | 2002, 2007, 2012 | Walker et al. 2012, Travers and Potter 2002, Jackson et al. 2007 |
| Lethrinus laticaudis | 2002, 2012 | Walker et al. 2012, Travers and Potter 2002 |
| Leviprora inops | 2002 | Travers and Potter 2002 |
| Monacanthus chinensis | 2002, 2012 | Walker et al. 2012, Travers and Potter 2002 |
| Lethrinus choerynch | 2007 | Jackson et al. 2007 |
| Lethrinus genivittatus | 2007 | Jackson et al. 2007 |
| Lethrinus laticaudis | 2007 | Jackson et al. 2007 |
| Lethrinus miniatus | 2007 | Jackson et al. 2007 |
| Lutjanus argentimaculatus | 2007 | Jackson et al. 2007 |
| Lutjanus vitta | 2007 | Jackson et al. 2007 |
| Orectolobus hutchinsi | 2012 | Walker et al. 2012 |
| Pagrus auratus | 2002, 2007, 2012 | Walker et al. 2012, Travers and Potter 2002, Jackson et al. 2007 |
| Parapercis nebulosa | 2002, 2007 | Travers and Potter 2002, Jackson et al. 2007 |
| Pelates quadrilineatus | 2002 | Travers and Potter 2002 |
| Pelates octolineatus | 2002, 2007, 2011, 2012 | Walker et al. 2012, Travers and Potter 2002, Bessey and Heithaus 2015, Jackson et al. 2007, Belicka et al. 2012, Heithaus et al. 2011 |
| Pelates quadrilineatus | 2012 | Walker et al. 2012 |
| Pentapodus vitta | 2002, 2007, 2012 | Walker et al. 2012, Travers and Potter 2002, Jackson et al. 2007, Belicka et al. 2012 |
| Platycephalus endrachtensis | 2002, 2007, 2012 | Walker et al. 2012, Travers and Potter 2002 |
| Priolepis semidoliatus | 2002 | Travers and Potter 2002 |
| Psammoperca waigiensis | 2002, 2007, 2012 | Walker et al. 2012, Travers and Potter 2002, Jackson et al. 2007 |
| Pseudorhombus arsius | 2002 | Travers and Potter 2002 |
| Pseudorhombus jenynsii | 2002 | Travers and Potter 2002 |
| Rhabdosargus sarba | 2007, 2011, 2012 | Walker et al. 2012, Jackson et al. 2007, Belicka et al. 2012, Heithaus et al. 2011 |
| Scaevius milii | 2012 | Walker et al. 2012 |
| Scobinichthys granulatus | 2002 | Travers and Potter 2002 |
| Siganus fuscescens | 2002, 2012 | Walker et al. 2012, Travers and Potter 2002 |
| Sillago vittata | 2012 | Belicka et al. 2012 |
| Synodus sageneus | 2002 | Travers and Potter 2002 |
| Torquigener pleurogramma | 2002, 2012 | Walker et al. 2012, Travers and Potter 2002 |
| Torquigener whitleyi | 2002, 2012 | Walker et al. 2012, Travers and Potter 2002 |
| Upeneus tragula | 2002, 2012 | Walker et al. 2012, Travers and Potter 2002 |
